# Integrating Psychosocial Care into Neuro-Oncology: Challenges and Strategies

**DOI:** 10.3389/fonc.2015.00041

**Published:** 2015-02-23

**Authors:** Suzanne K. Chambers, Luigi Grassi, Melissa K. Hyde, Jimmie Holland, Jeff Dunn

**Affiliations:** ^1^Griffith Health Institute, Griffith University, Brisbane, QLD, Australia; ^2^Cancer Council Queensland, Brisbane, QLD, Australia; ^3^Health and Wellness Institute, Edith Cowan University, Perth, WA, Australia; ^4^Prostate Cancer Foundation of Australia, Sydney, NSW, Australia; ^5^Centre for Clinical Research, University of Queensland, Brisbane, QLD, Australia; ^6^Institute of Psychiatry, Department of Biomedical and Specialty Surgical Sciences, University of Ferrara, Ferrara, Italy; ^7^Memorial Sloan Kettering Cancer Center, New York, NY, USA; ^8^School of Medicine, Griffith University, Brisbane, QLD, Australia; ^9^School of Social Science, University of Queensland, Brisbane, QLD, Australia

**Keywords:** neuro-oncology, psychosocial care, brain tumours, survivorship, distress

## Abstract

Approximately 256,000 cases of malignant brain and nervous system cancer were diagnosed worldwide during 2012 and 189,000 deaths, with this burden falling more heavily in the developed world. Problematically, research describing the psychosocial needs of people with brain tumors and their carers and the development and evaluation of intervention models has lagged behind that of more common cancers. This may relate, at least in part, to poor survival outcomes and high morbidity associated with this illness, and stigma about this disease. The evidence base for the benefits of psychosocial care in oncology has supported the production of clinical practice guidelines across the globe over the past decade, with a recent mandate to integrate the psychosocial domain and measurement of distress into routine care. Clinical care guidelines for people with brain tumors have emerged, with a building focus on psychosocial and survivorship care. However, researchers will need to work intensively with health care providers to ensure future practice is evidence-based and able to be implemented across both acute and community settings and likely within existing resources.

## The Health Burden of Brain Tumors

It was estimated that there were approximately 256,000 cases of malignant brain and nervous system cancer (ICD-10 codes C70-C72) diagnosed worldwide during 2012 (age standardized rate of 3.4/100,000) and 189,000 deaths (2.5/100,000) (1). The incidence rate of cancers of the brain and nervous system was almost double in more developed countries compared to less developed countries (5.1/100,000 and 3.0/100,000, respectively) and was higher for males (3.9/100,000) than females (3.0/100,000). Five-year prevalence was 343,000 in total. This disease carries a heavy psychosocial burden (2, 3), and often occurs at the age of middle adult life with 41% of brain tumor patients globally aged younger than 50 years (median age range of 55–59 years) (1). The middle adult life stage is a time of potential generativity (4), such that the loss of function and loss of life from an individual, family, community and economic perspective is substantial.

Patients with brain tumor suffer from a high rate of psychiatric and psychological disorders that are quite specific and distinct from other areas of psycho-oncology. In fact, unlike systemic effects of other tumors and treatment, brain tumors have a direct effect on brain functioning affecting cognition, mood, and personality, with profound changes in mood and cognition and impairments in several dimensions of functioning (5) and quality of life (6–8). A series of data have been collected regarding the effects of primary brain tumors on individual psychological functioning and psychosocial dimensions. The most significant and common disorders regard cognitive dysfunction, affecting about 70% of the patients. Disorders of memory, attention, and concentration have been described, with a tendency to worsen as the lesion increases or invades CNS areas. Acute confusional states (i.e., delirium) are also common neurocognitive complications of brain tumors. Clinically, some dysfunctions and symptoms are described in terms of “specific” syndromes, such as frontal lobe syndromes (caused by tumors in the frontal lobe) with several manifestations, including agitation, behavioral disruption and emotional lability (e.g., orbitofrontal disinhibited syndrome), psychomotor slowness and apathy (e.g., mesial frontal apathetic syndrome), and disorders of the executive functions, perseveration, and psychomotor slowing (e.g., dorsolateral prefrontal dysexecutive syndrome); temporal lobe syndrome, with impairment of verbal and non-verbal memory and seizures (9). A further major challenge of these disorders, and in neuro-oncology in general, is represented by a frequently undetected and under-recognized possible effect of psychiatric disorders, mainly cognitive impairment, in reducing patients’ mental capacity with problems in providing informed consent (10, 11).

Further syndromes related to brain tumors that have to be taken into account regard mood disorders, including depression and mania (25–30%), anxiety disorders (15–70%), changes in personality traits (sometimes subtle in the beginning phase, sometimes abrupt and dramatic), and psychotic disorders (12). Significant neuropsychiatric disorders may be the consequence of intervention, including surgery, radiotherapy, and, especially, drugs (e.g., psychotic syndromes and behavioral disorders secondary to corticosteroids) (13). Evaluation of patients’ symptoms, by conducting a careful neuropsychological and psychiatric assessment, is mandatory in clinical settings in order to provide the most proper psychopharmacological (e.g., antidepressants, anticonvulsants, antipsychotics) and psychotherapeutic intervention. With regard to the latter, the need for specific educational, supportive, and psychosocially oriented intervention for the patients’ families has also been repeatedly underlined (14–16). However, a recent review concluded that the research to date on the complex needs of brain tumor patients and how to best help them is limited in scope, with little attention to how to provide supportive care (17). This gap also extends to survivorship care and planning.

## Cancer Survivorship, Stigma, and Social Representations of Illness

The National Cancer Institute defines cancer survivorship as focusing on the health and life of a person with cancer from diagnosis and treatment until end of life, including the physical, psychosocial, and economic issues of cancer through the balance of his or her life. Within this definition, the experience of family members, friends, and caregivers are also considered relevant (18). The language applied within this discourse is intended to be empowering, signaling a shift from cancer “victim” terminology to a survivor framework. However, not all people who have had cancer perceive themselves to be a cancer survivor (19), and some suggest that this label marginalizes those who have a poor prognosis or high cancer-related morbidities (20, 21).

Stigma is when a person is seen by society as tainted, damaged, or less valuable as a result of an attribute or characteristic (22). Stigmatizing marks can be linked to appearance (e.g., physical appearance or overt behavioral differences) or group membership (e.g., race or religion), and is relationship and context specific (23). In health, stigma is reported to be higher for illnesses that are linked to modifiable lifestyle factors (e.g., smoking, drug or alcohol abuse, sexual activity), disfigurement or outward signs of illness, or a painful death (24). For example, cervical cancer has been reported as stigmatizing on the basis of its relationship with human papilloma virus and from this inferred sexual activity (25). People with lung cancer report feeling stigmatized based on the connection between smoking and lung cancer, as well as the high morbidity and mortality of the disease (24). The changes in facial appearance that may accompany head and neck cancer have been linked to stigma in this patient group (26) and patients with Parkinson’s disease who have facial masking are more negatively judged that those with normal expressivity (27). Finally, epilepsy is reported as being globally one of the most stigmatizing health conditions, linked to perceptions of it as being unpredictable, unattractive and violent, and representative of mental illness (28, 29).

Hence, although it is suggested that stigma about cancer in general has declined over the past four decades (30), some patient groups still experience stigma. People with brain tumors may experience stigma as a result of the cognitive, behavioral, and physical changes that may result from the tumor or treatment, as well as fears about a cancer that for some may have a poor outcome. Brain tumor patients therefore may experience social stigma as a result of their cognitive and neurological symptoms, and this may deepen these patients’ sense of social isolation and discrimination (31). Within this, the perception of a brain tumor as “mind-body” illness may be stigmatizing for both the patient and their family. In some cultures, this effect is worsened by lay beliefs about the causes of illness. For example, in a qualitative study in Bangalore, people with glioblastoma reported that their illness was a punishment from God for previous sins, or Karma, or a result of black magic (32). Palese et al. proposed that patients with frontal lobe neoplasms may be more at risk of stigma and having their problems underestimated by nurses than those with other cerebral neoplasms (33). However, findings were mixed with a tendency for nurses to overestimate problems more common. It is perhaps surprising, however, how little research has been undertaken about health-related stigma in brain tumor patients and how this affects their lives and their access to and utilization of health care services.

In this regard, stigma is connected to poorer outcomes in life across the domains of health, education, and access to social resources and in the case of people with stigmatized health conditions contributes to higher subjective distress about their illness (34). It is well accepted that there is a stigma around mental illness in Western culture (35, 36), and it has been further suggested that this stigma is also a barrier to cancer patients seeking and obtaining help for the distress associated with cancer (37). This means that patients who have a stigmatizing cancer may be doubly disadvantaged: more distress and less help. In addition, a broader health sector outcome of stigma [that has been well discussed in lung cancer (38)] is that stigmatized conditions may be underfunded for research and services. Consistent with this, in 2004 in the House of Commons John Brecow, the Chair of the brain tumor All Party Parliamentary Group made the point that “the issue of brain tumors is under-debated, under-reported, and under-funded. In this Parliament, the issue has attracted minimal – dare I say it, derisory – attention.” In this context, quality frameworks for health service delivery can play a crucial role in evening the playing field.

## Guidelines and Quality Standards for Psychosocial Care

Psycho-oncology and psychosocial oncology are, relative to biomedical treatments for cancer, a recent development in modern cancer care. Surgical treatment was the forerunner of cancer treatment, an approach that became more widely possible in the nineteenth century with the development of anesthesia and the first successful brain tumor surgical removal reported in 1879 (39). At the beginning of the twentieth century radiation therapy emerged as a cancer treatment (40), followed in 1940s by chemotherapy (41). By contrast, although the psychosocial care of people with cancer arguably does not hinge on technological advancement, the emergence of this field followed decades later, perhaps best heralded by the formation in 1984 of the International Psycho-Oncology Society (IPOS). IPOS led the mission to improve the care of cancer patients and their families globally by promoting the science of psycho-social and behavioral oncology (42) and the publication in 1989 of the first textbook in the field (43). A more recent milestone was the introduction of quality standards for psychosocial care by IPOS in 2010 (44). Parallel to these developments was the emergence of the cancer survivorship movement, with the formation in 1986 of the National Coalition for Cancer Survivorship (NCCS). The NCCS promoted itself as an advocacy collective for cancer survivors followed a decade later by the National Cancer Institute Office of Cancer Survivorship with the mission to promote cancer survivorship programs and research.

Over the past decade, a number of countries have developed generic clinical practice guidelines for the psychosocial care of adults with cancer. In Australia, these were first developed for women with breast cancer, and then later revised in 2003 to cover all adults with cancer (45). Similar work followed after in Canada, United Kingdom, and European Union (46–48). However, while clinical practice guidelines provide an evidence-based reference point to guide care, they are limited by the *a priori* review scope and are of less direct application in a field where evidence is scant. This means that the depth of direction and advice to addressing the specific and specialized needs of brain tumor patients and their families in such guidelines is limited. As well, the development and dissemination of guidelines do not necessarily change practice (49). Further actions to improve practice in psychosocial care included the Institutes of Medicine 2007 report “Cancer Care for the Whole Patient: Meeting Psychosocial Health Needs” with the major recommendation that “quality cancer care today must integrate the psychosocial domain into routine cancer treatment” (50). In 2010, IPOS published a new international quality standard supporting the integration of psychosocial care and proposing a distress screening and management be included in routine care by placing it as one of the six Vital Signs (44). These standards have now been widely endorsed internationally.

A number of medically focused guidelines specific to brain tumors have been developed in Australia, United Kingdom, and North America (34, 51–55). While broadly speaking these tend to focus on the medical management of diagnosis and treatment, the Australian Clinical Practice Guidelines for the Management of Adult Gliomas: astrocytomas and oligodendrogliomas addresses the cognitive and personality changes that can occur in these patients and provides recommendations for identification and management of psychological disorders, cognitive problems and personality, and other changes related to the tumor or its treatment (52). This includes advice about the need for early identification of psychological distress and referral for psychosocial treatment for those with or at risk of significant distress. Neuro-rehabilitation within a multi-disciplinary care model is also advised. This approach of psychological assessment and support as an integral part of the management of patients with brain tumor is also advised elsewhere with referral to neuropsychology and neuropsychiatry services advised for patients who require specialist intervention for cognitive, emotional, or behavioral problems (54). Nursing clinical practice guidelines developed by the American Brain Tumor Association and the American Association of Neuroscience Nurses also specifically address nursing assessment for a range of problems including fatigue, distress, and body image with referral for rehabilitation (51). Notably, these guidelines also discuss survivorship issues including the need for support for caregivers. Finally, the National Comprehensive Cancer Network survivorship guidelines do note that that cognitive impairment is prominent in survivors of primary central nervous system cancers or people with brain metastases; however, acknowledge that there is limited evidence to date to guide management of this condition, especially for cancers other than breast (53).

Despite these encouraging developments there are barriers to the implementation of psychosocial and survivorship care in oncology settings, which include the continued dominance of biomedical care models; gaps in knowledge about research translation; diminishing health budgets in the face of escalating costs; and individual and community attitudes to illness and help seeking (44, 56, 57). In brief, while quality standards and guidelines provide guidance for key characteristics of good oncology care, operationalizing these in the clinical or community setting presents its own challenges. Care models that are practically translatable are needed.

## Stepped Care Models

One approach to this problem has been to develop care frameworks that show how services articulate across levels of distress and that focus on delivering the most in-depth (and expensive) services to those who need them most. A tiered approach tailors services to need through screening, triage and referral to different levels of intervention appropriate to each patient (58). At the most basic level, psychosocial care would include cancer-related information and brief support from a health care professional in the treatment team; cancer-related telephone helpline and other information focused interventions. Those with higher levels of distress that require more specific psychological interventions, including people with pre-existing vulnerabilities or complex problems (e.g., neurocognitive deficits) are referred to more intensive, specialized, or multidisciplinary approaches. Transition to more specialized and in-depth levels of care is guided by standardized distress screening, as per the best practice internationally, and interview assessment by the treating health professional. A stepped care approach differs in that a decision analytic approach is applied with systematic identification of high need patients followed by an integrated treatment program where care is stepped up progressively until the problem is resolved (59). These approaches have not yet to our knowledge been applied to people with brain tumors; however, the articulation of a tiered or stepped care model for this patient group that incorporates specific needs of brain tumor patients seems warranted.

All such models are predicated on applying screening for distress to guide referral to the appropriate level of care, or stepping up of care as needed. The distress thermometer is an ultra-brief screening measure that has been widely validated globally across cultures and tumor sites and found to be a reliable first-line screening tool for detecting psychological distress in cancer patients (60, 61). This measure includes a problem checklist and a single item asking the patient how much distress they have been experiencing in the past week including the current day on a scale of 0, no distress to 10, extreme distress (62, 63). Although the most commonly recommended cut-off for this scale is >4, in the case of people with intracranial tumors a cut-off of >6 has been reported as having optimal sensitivity for detecting distress (64). A key advantage of the distress thermometer is that it is short and easy to administer and score thus making it ideal for translation into acute settings. Other researchers have found the two item Patient Health Questionnaire-2 (65) to have acceptable psychometric properties for detecting moderate to severe psychological distress in brain tumor patients (66). In contrast, Rooney et al. (67) have recommended longer scales and in particular the Hospital Anxiety and Depression Scale (68) and Patient Health Questionnaire-9 (69) for detecting major depressive disorder in well-functioning glioma patients as a preceding step to more in-depth clinical assessment (67). The important question of how screening for neurological and cognitive impairment can be undertaken in these patients alongside distress screening, particularly in settings where specialist staff may not be easily accessed, is a key future question for both researchers and health care providers.

## Conclusion

**Figure 1:**
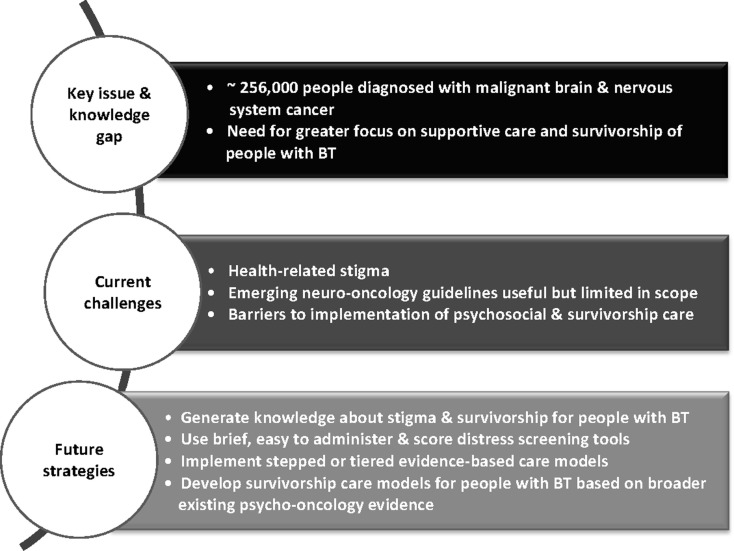
**Overview of challenges and strategies for integrating psychosocial care into neuro-oncology**.

There is a need for a comprehensive model of survivorship care for people affected by brain tumor and their families and this should be a priority for neuro-oncology (Figure 1). Given the more advanced stage of development of such care in other cancers, there is a platform of existing knowledge upon which neuro-oncology practitioners may build. This includes screening for distress with referral as needed into stepped and evidenced-based care models. However, although clinical care guidelines specifically for people with brain tumors are emerging, there is a scarcity of intervention research in the field. There is a clear need for a strategic focus on knowledge generation around survivorship for this patient group.

## Conflict of Interest Statement

The authors declare that the research was conducted in the absence of any commercial or financial relationships that could be construed as a potential conflict of interest.
